# Quality of life in patients with liver tumors treated with holmium-166 radioembolization

**DOI:** 10.1007/s10585-019-10006-1

**Published:** 2019-11-15

**Authors:** Caren van Roekel, Maarten L. J. Smits, Jip F. Prince, Rutger C. G. Bruijnen, Maurice A. A. J. van den Bosch, Marnix G. E. H. Lam

**Affiliations:** Department of Radiology and Nuclear Medicine, University Medical Center Utrecht, Utrecht University, Heidelberglaan 100, 3584 CX Utrecht, The Netherlands

**Keywords:** Radioembolization, Holmium-166, Quality of life, Hepatic metastases

## Abstract

Holmium-166 radioembolization is a palliative treatment option for patients with unresectable hepatic malignancies. Its influence on quality of life has not been evaluated yet. Since quality of life is very important in the final stages of disease, the aim of this study was to evaluate the effect of holmium-166 radioembolization on quality of life. Patients with hepatic malignancies were treated with holmium-166 radioembolization in the HEPAR I and II studies. The European Organization for Research and Treatment of Cancer QLQ-C30 and LMC21 questionnaires were used to evaluate quality of life at baseline, 1 week, 6 weeks and at 6, 9 and 12 months after treatment. The course of the global health status and symptom and functioning scales were analyzed using a linear mixed model. Quality of life was studied in a total of 53 patients with a compliance of 94%. Role functioning was the most affected functioning scale. Fatigue and pain were the most affected symptom scales. Changes in almost all categories were most notable at 1 week after treatment. A higher WHO performance score at baseline decreased global health status, physical functioning, role functioning and social functioning and it increased symptoms of fatigue, dyspnea and diarrhea. Quality of life in salvage patients with liver metastases treated with holmium-166 radioembolization was not significantly affected over time, although a striking decline was seen during the first week post-treatment. A WHO performance score > 0 at baseline significantly influenced quality of life.

**Electronic supplementary material:**

The online version of this article (10.1007/s10585-019-10006-1) contains supplementary material, which is available to authorized users.

## Background

Radioembolizaton (RE) is an intra-arterial therapeutic option for patients with unresectable hepatic malignancies. Tumors within the liver receive their blood supply almost entirely from the hepatic artery whereas the normal liver is supplied mainly from the portal vein. Therefore, infusion of radiolabeled microspheres into the arterial system results in delivery of effective doses of radiation to the tumor without causing intolerable toxicity to the normal liver [1].

Holmium-166-poly(L-lactic acid) (^166^Ho)-microspheres (QuiremSpheres^®^, Quirem Medical B.V., The Netherlands) have been developed as an alternative to yttrium-90 (^90^Y) microspheres. The main advantage of ^166^Ho-microspheres is the ability to be visualized in vivo by SPECT and MRI, which enables quantitative biodistribution imaging [2]. ^166^Ho-microspheres have a mean diameter of 30 µm (range 15–60 µm). Overall, RE is safe and well tolerated, with primarily short-term toxicity. Mild clinical side effects of RE consist mainly of abdominal pain, nausea, vomiting, fatigue and fever and usually occur within 4–6 weeks after treatment (post-embolic syndrome) [3, 4]. Palliative chemotherapy in the same setting, however, is known to be associated with substantial side effects [5]. With the advances in cancer treatment and increased survival, quality of life (QoL) has become increasingly important [6]. Tumor-specific therapy can potentially prolong life, but, due to its possible toxicity, may considerably reduce QoL [7]. The majority of patients (82–95%) value the impact on QoL of the treatment at least as much as the survival benefit [8, 9]. Factors known to influence QoL in cancer patients are, among others, age, gender, cancer type, performance status, and high symptom burden [10–13]. In patients with hepatic malignancies, specifically, extrahepatic recurrence is of significant influence on QoL [14]. To form an impression of the influence of RE on QoL, we performed a systematic review of the literature (See Figure S1 for the search strategies). The effect of Y90-RE on QoL was investigated in 14 studies [15–28]. In most studies, QoL did not change significantly after Y90-RE (Table 1) [15, 17, 19–21, 23, 25, 27]. In a minority, QoL either improved [16, 26] or worsened after ^90^Y-RE [18, 24]. The purpose of the current study was to evaluate the effect of ^166^Ho-RE on QoL. Based on the literature, our hypothesis was that QoL would not be significantly affected by ^166^Ho-RE, similar to what is known for ^90^Y-RE. Furthermore, the hypothesis was that QoL may be impaired by the known short-term side-effects of ^90^Y-RE, i.e. the post-embolization syndrome.

**Table 1:** Overview of literature

Solely Y-RE	First author, year	Treatment arm	Control arm	n (Y-RE/other)	Primary tumor(s)	RE approach	Questionnaires	Scale range	Timing	Outcome
	Cosimelli et al. [15]	Y-RE	–	14^a^	Colorectal	Whole liver, re–RE in 3 patients	QLQ-C30, QLQ-LMC21, QLQ-CR38	0–100	Baseline, 6 weeks	QoL was not adversely affected
	Kalinowski et al. [16]	Y-RE	–	9	Neuroendocrine tumour	7 patients whole liver, 2 patients bilobar with re–RE	QLQ-C30, QLQ-LMC21	0–100	Baseline, 3-monthly (up to 44 months)	After 6 months, QoL significantly improved
	Salem et al. [17]	Y-RE	TACE^b^	29/27	HCC^c^	20 patients lobar, 9 patients segmental	Fact-Hep	0–180	Baseline, 2 weeks, 4 weeks	No significant difference between arms
	Steel et al. [18]	Y-RE	TACE	14/14	HCC	Whole liver	Fact-Hep	0–180	Baseline, 3 months, 6 months, 1 year	At 3 months, significantly higher QoL scores for Y-RE group than control group. No significant difference at 6 months
	Kolligs et al. [19]	Y-RE	TACE	8/10^d^	HCC	5 patients lobar, 1 patient segmental, 7 patients whole liver	Fact-Hep	0–180	Baseline, 6 weeks, 12 weeks	No significant difference between groups
	Cramer et al. [23]	Y-RE	–	30	Neuroendocrine tumour	Lobar	Short Form-36 Health Survey Form	0–100	Baseline, 1,3,6,12,24 months	QoL was sustained for up to 24 months following treatment
	Vilgrain et al. [28]	Y-RE	Sorafenib	184/206	HCC	205 lobar treatments, 81 segmental/sector treatments	QLQ-C30, EORTC-HCC18	0–100	Baseline, 1 month, 3-monthly (up to 12 months)	Global health status was significantly better in the Y-RE group than in the sorafenib group
	Kirchner et al. [25]	Y-RE	TACE	21/46	HCC	NR	QLQ-C30, EORTC-HCC18	0–100	Baseline, 2 weeks	QoL was not significantly affected and there was no significant difference between groups
	Gill et al. [26]	Y-RE	TACE, sorafenib		HCC	NR	Online survey	NR	NR	QoL improved after RE and TACE compared to sorafenib
	Xing et al. [27]	Y-RE	–	30	HCC	Lobar	Short Form-36 Health Survey Form	0–100	Baseline, 1, 3, 6 months	No significant changes in QoL
Y-RE + chemo	Gray et al. [20]	Y-RE & 5-FU	5-FU^e^	36/34	Colorectal	Whole liver	Self Assessment Scale		Baseline, 3-monthly (up to 18 months)	No significant difference, in both arms QoL tended to improve
	Van Hazel et al. [21]	Y-RE & 5-FU/LV	5-FU/LV^f^	11/10	Colorectal	Whole liver	FLIC questionnaire, Spitzer index		Baseline, 3-monthly (up to 36 months)	No significant difference between arms
	Chow et al. [22]	Y-RE & sorafenib	–	29	HCC	20 patients whole liver, 9 patients lobar	EQ-5D Index		Baseline, every month until progression, 6-month intervals after progression	EQ-5D index in BCLC^g^ stage B decreased over time, while it increased in BCLC Stage C
	Wasan et al. [24]	Y-RE & FOLFOX	FOLFOX	554/549	Colorectal	NA^h^	EQ-5D-3L Index	0–1	Baseline, 2-3,6,12,24 months	EQ-5D-3L index decreased over time in both groups, no clinically meaningful differences

^a^Of 50 included patients, 14 were evaluated for QoL

^b^Transarterial chemoembolization

^c^Hepatocellular carcinoma

^d^10 patients with missing baseline data were excluded from QoL analysis

^e^5-Fluorouracil

^f^Leucovorin

^g^Barcelona Clinic Liver Cancer

^h^Not available

## Materials and methods

### Patients and study design

QoL was evaluated in patients included in the HEPAR I and HEPAR II studies (clinicaltrials.gov identifier NCT01031784 and NCT01612325). The inclusion criteria for treatment were exactly the same and the patient population in both studies was comparable (Table S1). In these studies, patients with unresectable, chemorefractory liver metastases of any primary origin and cholangiocarcinoma were included. Patients were eligible if they were diagnosed with liver-dominant disease, had a life expectancy of > 3 months, had measurable disease on CT, had adequate liver, renal and bone marrow function, and had a WHO performance score of ≤ 2. The institutional review board approved these studies and all patients provided written informed consent. The aim of the HEPAR I study was to assess the safety and the maximum tolerated radiation dose of ^166^Ho-RE. The maximum tolerated dose was found to be 60 Gy and its safety and efficacy was established in the HEPAR II study. A more detailed description of the study designs and the main study results have been published elsewhere [29–31].

### Treatment

Patients received a work-up angiography approximately 1 week before treatment in which extra-hepatic vessels were coil-embolized, if necessary. A scout dose of ^99m^Tc-MAA (150 MBq, Technescan LyoMAA^®^; Mallinckrodt Medical B.V., Petten, The Netherlands) was administered to assess the extrahepatic and intra-hepatic distribution. After a 1–2 week interval, patients were scheduled for a second and third angiography. The second angiography was planned in the morning, during which patients received a scout dose of ^166^Ho-microspheres, directly followed by SPECT and MRI. The treatment dose of ^166^Ho-microspheres was administered that same afternoon and was followed by SPECT and MR image acquisition 3–5 days later [30, 31].

### Quality of life assessment

QoL in patients was assessed using the validated European Organization for Research and Treatment of Cancer (EORTC) QLQ-C30 version 3.0 and QLQ-LMC21 questionnaires [32] [33]. The EORTC QLQ-C30 contains 30 questions and the EORTC QLQ-LMC21 contains 21 items. They are composed of both multi-item scales and single-item measures: from the questionnaires, a Global Health Status/Quality of Life (GHS), 5 functioning scales and 22 symptom scores were derived. All but two items are scored on 4-point Likert scales (1: not at all, 2: a little, 3: quite a bit, 4: very much). The two other items are scored on a 7-point linear analogue scale. The raw subscale scores are transformed to a 0–100 scale, where a high score in a functioning scale represents unimpaired functioning and a high score in a symptom scale represents a high level of symptomatology. The functioning scales are: physical functioning (PF), role functioning (RF), emotional functioning (EF), cognitive functioning (CF) and social functioning (SF). The symptom scales are: fatigue (FA), nausea and vomiting (NV), pain (PA), dyspnea (DY), insomnia (SL), appetite loss (AP), constipation (CO), diarrhea (DI), financial difficulties (FI)(QLQ-C30); and eating (LMNutri), fatigue (LMCFati), pain (LMCPA), emotional problems (LMCEp), weight loss (LMCWL), taste (LMCTA), dry mouth (LMCDM), sore mouth/tongue (LMCSM), peripheral neuropathy (LMCPN), jaundice (LMCJ), contact with friends (LMCFr), talking about feelings (LMCFeelings), and sex life (LMCSx) (QLQ-LMC21).

Patients received the questionnaires at baseline, 6 weeks and 3 months after treatment. Follow-up in the HEPAR II study was longer, so those patients also received the questionnaires at 6, 9 and 12 months after treatment. The last included 26 patients of the HEPAR II study received an extra questionnaire 1 week after treatment to better reflect patients’ transient symptoms shortly after treatment [30, 31].

### Response assessment

Response assessment was based on contrast-enhanced CT at 3 months posttreatment, according to the Response Evaluation Criteria in Solid Tumours (RECIST) version 1.1 [34].

#### Scoring and statistical analysis

Scoring of the questionnaires was performed according to the scoring manual provided by the EORTC (EORTC scoring manual). Missing values were imputed using multiple imputation. Internal consistency of the multi-item scales was determined using Cronbach’s alpha.

Kolmogrov-Smirnov and Shapiro–Wilk tests were carried out for all categories at the different time points and showed that the data were not normally distributed (p ≤ 0.001).

Descriptive analyses were performed to summarize patient demographics and treatment characteristics. A linear mixed-effects regression model was fitted to evaluate the development of QoL, taking into account all available data [35]. The influence of the following variables on QoL was tested, as these were believed to be of possible influence on QoL: gender (male versus female), previous treatments (systemic, locoregional, both or none), extrahepatic disease at baseline (yes/no), performance status at baseline (WHO score 0, 1 or 2), primary tumor type (colorectal carcinoma versus other), time and response category (complete response, partial response, stable disease or progressive disease). Random effects were tested based on Akaike’s information criterion and fixed effects were tested using a backward stepwise approach.

A relatively conservative *P* value ≤ 0.001 (instead of ≤ 0.05) was considered statistically significant in order to reduce type I errors [36]. Statistical analyses were performed using R (version 3.5.1).

## Results

QoL was studied in a total of 53 patients treated with ^166^Ho-RE between November 2009 and March 2015; 15 patients in the HEPAR I study and 38 patients in the HEPAR II study (Flowchart for study inclusions: Figure S2). Patient characteristics are listed in Table 2.

**Table 2:** Baseline characteristics of treated patients in the HEPAR I and II studies

Characteristic	Value
N	
	53
Age (years)	
Median (range)	66 (38–87)
Gender	
Male (%)	31 (58%)
Primary tumour—no.	
Colorectal	29
Ocular melanoma	8
Cholangiocarcinoma	6
Breast carcinoma	5
Neuroendocrine tumour	2
Pancreatic cancer	1
Gastric cancer	1
Thymoma	1
Administered activity (MBq)	
Median (range)	6210 (1615–13187)
Aimed whole liver dose (Gray)—no.	
20	6
40	3
60	41
80	3
Previous therapies	
Systemic treatment	43
Locoregional treatment	10
Treatment procedure	
Whole liver	48
Lobar	5
WHO performance status	
0	45
1	7
2	1
Extrahepatic metastases	
Bone	4
Lung	9
Lymph node	8
None	33

Baseline characteristics of patients treated with ^166^Ho-RE in the HEPAR I and II studies

Due to the dose-escalating nature of the HEPAR I study, 9 patients received an aimed whole liver dose < 60 Gy (i.e. 20 Gy [n = 6], 40 Gy [n = 3]). The other 44 patients received an aimed whole liver dose of ≥ 60 Gy. One patient was excluded from response analysis because this patient did not receive contrast at 3-month follow-up CT-scan. Based on 3-month follow-up CT (using the RECIST 1.1 evaluation), 8 patients had partial response and 14 patients had stable disease. The remaining 28 patients had progressive disease.

### Compliance

Fifty of 53 patients (94%) filled out the baseline questionnaire and at least 1 follow-up questionnaire. Since patients were withdrawn from the HEPAR II study after diagnosis of progressive disease, there was quite some variability in follow-up time. Three patients failed to fill out the questionnaire at baseline and 3 months after treatment and were therefore excluded from analysis. Three patients failed to fill out a follow-up questionnaire (1 patient at 6 weeks and 2 patients at 6 months after treatment) and these questionnaires were pairwise excluded from analysis. Four patients left a question blank.

### Development of QoL

**Fig. 1:**
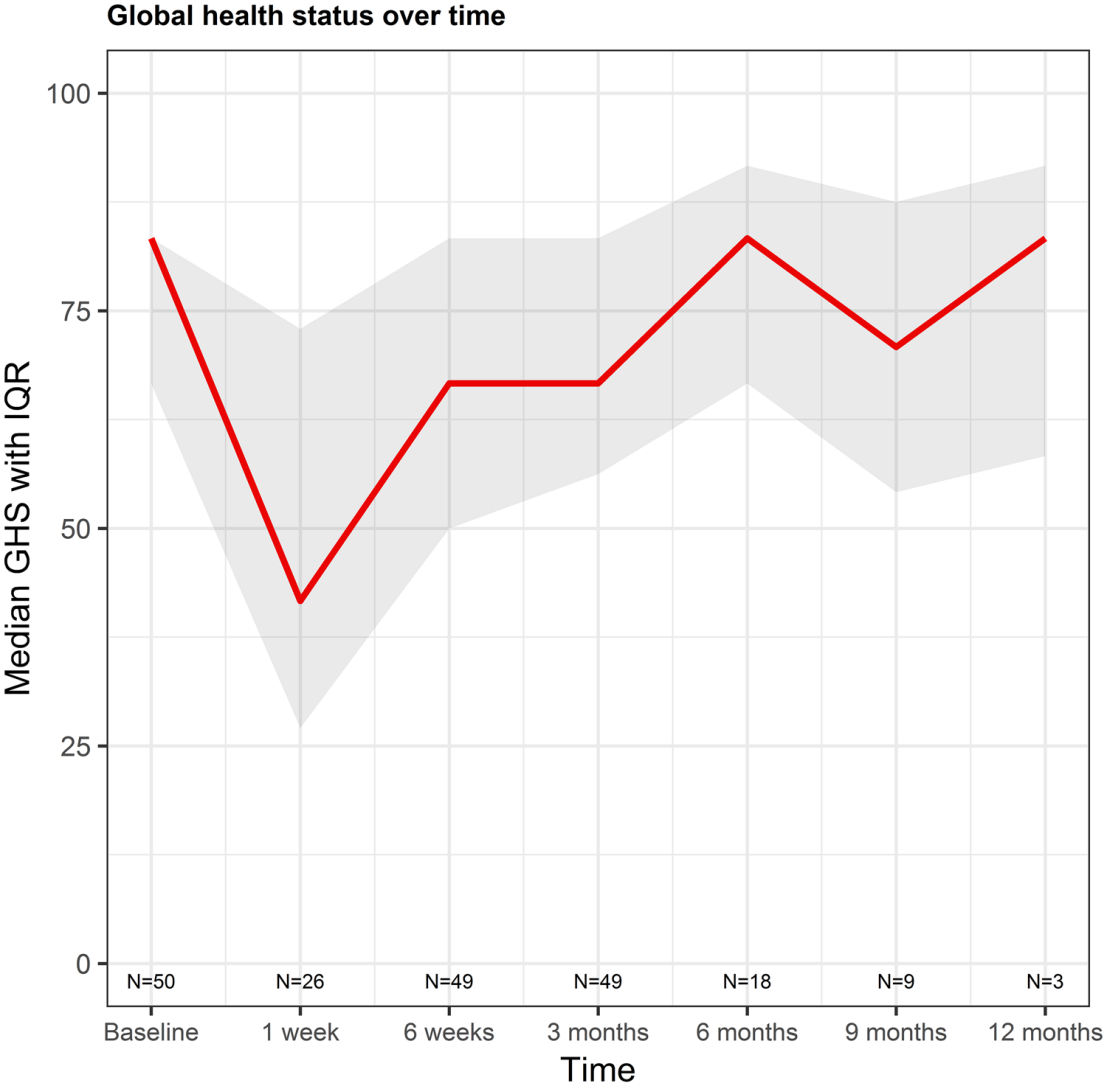
Median global health score over time with interquartile range (shaded area). A high score represents a good health score

**Fig. 2:**
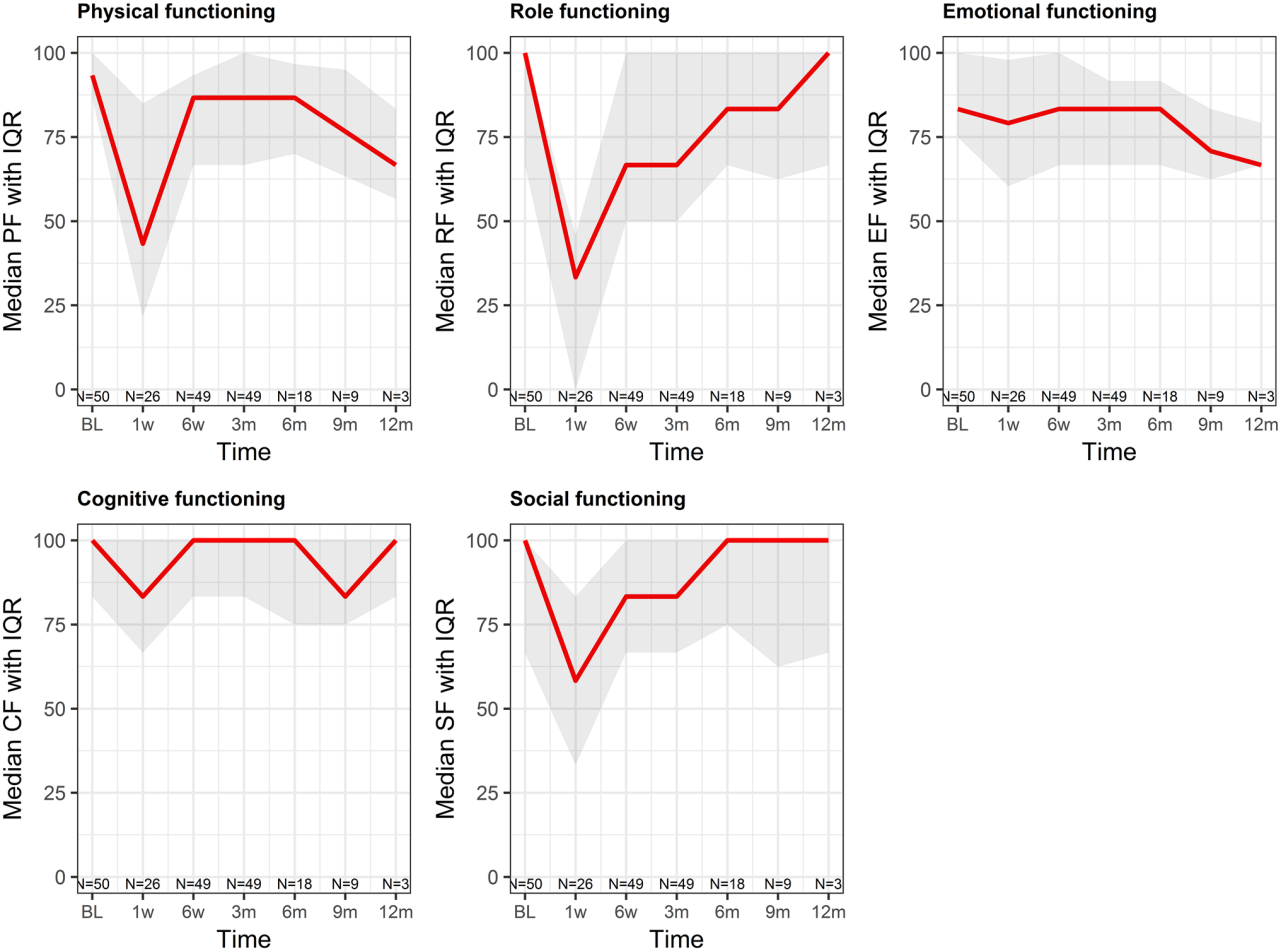
Median role functioning scores over time with interquartile ranges (shaded areas). *BL* baseline, *1w* 1 week, *6w* 6 weeks, *3* *m* 3 months, *6* *m* 6 months, *9* *m* 9 months, *12* *m* 12 months. A high score represents good functioning

Median and interquartile ranges of all categories at the different time points are listed in table S3 and graphically displayed in Figs. 1 and 2 and supplemental figure S3a-d. Cronbach’s alpha was determined for the multi-item scales at baseline and at 3 months follow-up and varied from 0.52 to 0.95 (Table S2).

From the figures it can be depicted that changes in almost all categories were most notable at 1 week after treatment. Role functioning was the most affected functioning scale. Fatigue and pain were the most affected symptom scales. Although there were very few patients that filled in the questionnaires beyond 3 months follow-up, all categories seemed to stabilize over time. At every time point, there was a lot of variation between patients in all categories except FI, LMCSM, LMCJ and LMCFeelings.

The development of QoL was best explained by a linear mixed-effects regression model using a random intercept per patient, to allow for different starting points at baseline.

**Fig. 3:**
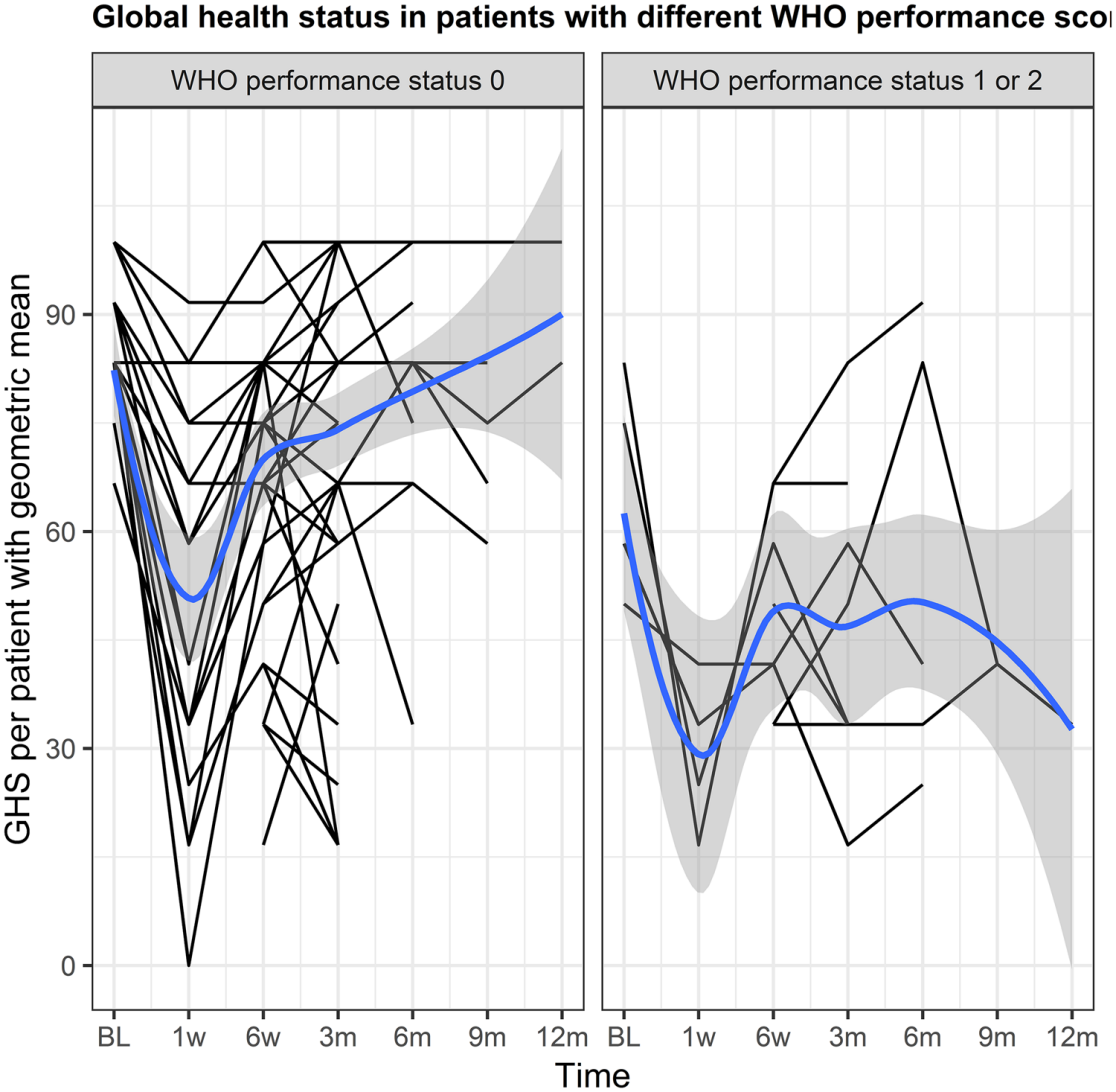
Global health status in patients with different WHO performance scores. The black lines depict the development of GHS per patient. The blue lines with shaded area represent the geometric mean with standard deviation. *BL* baseline, *1w* 1 week, *6w* 6 weeks, *3* *m* 3 months, *6* *m* 6 months, *9* *m* 9 months, *12* *m* 12 months. (Color figure online)

For GHS, as a general measure of quality of life, an increase of on average 0.55 points per time point was found. However, this was not significant (p = 0.48) and there was quite some variation between patients, as can be seen in Fig. 1. Still, there was a steep decline in functioning scores and rise of symptoms from baseline to 1 week. Patients with a higher WHO performance score had on average 20 points lower GHS (p = 0.0002, 95% CI [− 32.3;− 8.8]). No other variables were of significant influence on the development of GHS. Figure 3 shows the development of GHS per patient for patients with WHO performance scores of 0 versus scores 1 or 2. Although there is a lot of variation between patients, patients with a lower WHO performance score have on average a higher QoL.

In functioning scales, PF, RF and SF were significantly influenced by WHO performance status, where a higher WHO performance status at baseline decreased functioning (p < 0.001 in all categories).

In symptom scales, a higher WHO performance status increased mean symptom scores of FA, DY, DI, and LMCFati (p < 0.001 in all categories). There were no other variables that had a significant influence on the various symptom scores. Both within and between patients, there was a lot of variation in scores.

## Discussion

The purpose of the current study was to evaluate the effect of ^166^Ho-RE on QoL. The hypotheses were that there would be no significant change in QoL over time and that the post-embolization syndrome would have an impact on QoL. This study showed that the first hypothesis was correct: QoL was not significantly affected over time, although there was a lot of variation between and within patients. Regarding the second hypothesis; a decline in QoL and a rise of symptoms was seen at 1 week post-treatment, which is most likely due to the post-embolization syndrome, however, this was not statistically significantly different from the scores at baseline. In the linear mixed model analysis, it was shown that a higher WHO performance score significantly influenced PF, RF, SF, FA, DY, DI and LMCFati. This is not surprising, as patients with a higher WHO performance score are known to be in a debilitating physical condition, which likely influences their QoL.

There were no other variables that had a significant influence on QoL.

The GHS score was used as a general measure of QoL and is based on 2 questions. The other 49 questions (i.e. functioning and symptom scores) provide further insights why GHS changed. In this study, role functioning and social functioning were the most affected functioning scales. Role functioning is based on the patient’s ability to perform hobbies or other daily activities. Social functioning is measured to establish if one’s family life and social activities are influenced. Factors other than the treatment itself may influence these scores. Social functioning may for instance be affected by the instructions for radiation safety: all RE patients are instructed to keep a safe distance from family and relatives for the first days after treatment. In addition, participation in a clinical study with intensive monitoring and follow-up visits poses a significant time, psychological and physical burden, which may be reflected in decreased role- and social functioning. For the symptom scores, there was a rise in fatigue, pain, appetite loss, eating and contact with friends. The latter is coherent with social functioning. The prominent rise in pain and fatigue symptom scores is in accordance with the well-known side effects of RE: clinical side effects usually occur within the first 4 to 6 weeks after treatment and may consist of abdominal pain, nausea, vomiting, fatigue and slight fever [3].

In a subset of 26 patients, QoL assessment was added at 1 week post-treatment because it was thought this would better reflect the short-term adverse effects of the treatment. The steep decline in functioning scores and the rise of symptoms from baseline to 1 week is striking. This may be explained by the so-called post-embolization syndrome, which is known to occur after embolization therapies [3, 4, 37]. Future interventional oncology studies are encouraged to evaluate QoL shortly after treatment (i.e. < 2 weeks).

Due to a large number of differences between the available studies on QoL in patients treated with ^90^Y-RE and the HEPAR studies, such as the use of different questionnaires, different timing of the QoL evaluations and concomitant treatment with chemotherapy (Table 1), it is impossible to make a fair comparison. Only three studies studied QoL in patients treated with RE as a monotherapy, whereas the others studied RE in combination or in comparison with other therapies. Moreover, in the HEPAR studies, all patients received a whole-liver approach in a single session. This is a more aggressive treatment approach of RE and may have influenced QoL.

A higher number of ^166^Ho- and ^90^Y-resin microspheres (somewhere between 30–50 million) are typically injected for treatment in comparison with glass microspheres (typically several million). ^166^Ho- and ^90^Y-resin microspheres will therefore have a larger embolic effect and likely also more post-embolic symptoms such as pain, fever and loss of appetite. The study of Cosimelli et al. is most comparable to the HEPAR I and II studies. Cosimelli et al. reported that QoL was not adversely affected in their cohort of patients with metastatic colorectal carcinoma. However, QoL was not tested shortly after treatment, which is an important difference [15].

The changes in QoL after RE were also investigated in a first-line setting. In the SIRFLOX, FOXFIRE and FOXFIRE-Global studies, the possible role for RE as a first-line treatment was investigated. QoL was assessed in the patient group receiving systemic therapy alone and in the patient group receiving RE as an addition to systemic therapy. QoL was slightly worse in the combination group at 2-3 months follow-up, but this was not deemed clinically meaningful [24].

There are several limitations to this study. First, the total number of patients was limited. Second, there was a large loss to follow-up since patients were excluded from the HEPAR II study after diagnosis of progressive disease. This may also have led to a biased representation of the QoL of our study population and it may explain why response category did not significantly influence QoL in the analyses. Third, the QLQ-LMC21 questionnaire, created for patients with colorectal liver metastases, was used to complement the more general QLQ-C30 questionnaire, although colorectal cancer was not the only tumor type in this study. One of the strengths of this study is its prospective nature and the high compliance rate regarding the QoL questionnaires. QoL was frequently assessed and especially the 1-week post treatment questionnaire offered valuable insight in the short-term effects on QoL and patients’ transient symptoms. Another strength of this study is the use of a longitudinal approach for the data analysis. By using a mixed model with a random intercept per patient, the variation between patients and data clustering were taken into account.

More knowledge on the influence of ^166^Ho-RE on QoL is important for several reasons. Above all, this information is needed to better inform patients on treatment-related adverse effects and may help them to make a well-informed choice between all the available palliative treatment options. In selected populations, such as older patients or patients with multiple comorbidities, QoL is largely maintained. This can be a reason to prefer RE over other treatment modalities [28]. Furthermore, since RE is becoming more important in the first- and second-line settings, the impact of this therapy on QoL is also becoming more significant.

## Conclusion

In conclusion, QoL in salvage patients with liver metastases treated with ^166^Ho-RE was not significantly affected over time, apart from a decline during the first week after treatment. Changes in QoL were most notable during the first week post-treatment, probably due to the post-embolization syndrome. A WHO performance score > 0 at baseline significantly influenced QoL. Knowledge of the influence on quality of life of ^166^Ho-RE is important for patients to make a deliberate choice between palliative treatment options.

## Electronic supplementary material

Below is the link to the electronic supplementary material.

Supplementary material 1 (DOCX 19 kb)

Supplementary material 2 (XLSX 65 kb)

Search strategy for literature review of quality of life studies in patients treated with RE (TIFF 39490 kb)

Flowchart of included patients in the HEPAR I and II studies (TIFF 54 kb)

a–d Median symptom scores over time with interquartile ranges (shaded areas). BL = baseline, 1w = 1 week, 6w = 6 weeks, 3m = 3 months, 6m = 6 months, 9m = 9 months, 12m = 12 months. A high score represents a high level of symptomatology (TIFF 202500 kb)

Supplementary material 6 (TIFF 202500 kb)

Supplementary material 7 (TIFF 202500 kb)

Supplementary material 8 (TIFF 202500 kb)

## Data Availability

The dataset that supports the findings of this study is provided as supplementary material.

## Abbreviations

^90^Y: Yttrium-90
^99m^Tc-MAA: Technetium ^99m^Tc macro-aggregated albumin
^166^Ho: Holmium-166
AP: Appetite loss
CF: Cognitive functioning
CO: Constipation
DI: Diarrhoea
DY: Dyspnoea
EF: Emotional functioning
EORTC: European Organization for Research and Treatment of Cancer
FA: Fatigue
FI: Financial difficulties
GHS: Global health status
HCC: Hepatocellular carcinoma
LMCDM: Dry mouth
LMCEp: Emotional problems
LMCFati: Fatigue
LMCFeelings: Talking about feelings
LMCFr: Contact with friends
LMCJ: Jaundice
LMCPA: Pain
LMCPN: Peripheral neuropathy
LMCSM: Sore mouth/tongue
LMCSx: Sex life
LMCTA: Taste
LMCWL: Weight loss
LMNutri: Eating
MRI: Magnetic resonance imaging
NV: Nausea and vomiting
PA: Pain
PF: Physical functioning
QoL: Quality of life
RE: Radioembolization
RECIST 1.1: Response Evaluation Criteria In Solid Tumours
RF: Role functioning
SF: Social functioning
SL: Insomnia
SPECT: Single photon emission computed tomography
WHO: World health organization
