# Supplementary material for: Quality of life in patients with liver tumors treated with holmium-166 radioembolization
Source: Clin Exp Metastasis. 2019 Nov 15;37(1):95–105. doi: 10.1007/s10585-019-10006-1 (PMC7007912; doi:10.1007/s10585-019-10006-1)
Supplement: Supplementary file 1 — Supplementary material 1 (DOCX 19 kb) [file 10585_2019_10006_MOESM1_ESM.docx]

| Table S1. Most important characteristics of the HEPAR I and HEPAR II patient population | | | |
| --- | --- | --- | --- |
| **Characteristic** |  | **HEPAR I (n=15)** | **HEPAR II (n=38)** |
| Age (median, range) |  | 55 (38-87) | 66 (41-84) |
| Gender (n, %) |  |  |  |
|  | Male | 9 (60%) | 22 (58%) |
|  | Female | 6 (40%) | 16 (42%) |
| WHO performance status (n, %) |  |  |  |
|  | 0 | 13 (87%) | 32 (84%) |
|  | 1 | 2 (13%) | 5 (13%) |
|  | 2 | 0 (0%) | 1 (3%) |
| Primary malignancy (n, %) |  |  |  |
|  | Colorectal | 6 (40%) | 23 (61%) |
|  | Breast | 1 (7%) | 4 (11%) |
|  | Cholangiocarcinoma | 2 (13%) | 4 (11%) |
|  | Neuroendocrine tumor | 0 (0%) | 2 (5%) |
|  | Uveal melanoma | 6 (40%) | 2 (5%) |
|  | Pancreas carcinoma | 0 (0%) | 1 (3%) |
|  | Gastric cancer | 0 (0%) | 1 (3%) |
|  | Thymoma | 0 (0%) | 1 (3%) |
| Extrahepatic disease at baseline (n,%) |  |  |  |
|  | Yes | 6 (40%) | 10 (26%) |
|  | No | 9 (60%) | 28 (74%) |

| Table S2. Internal consistency of the multi-item scales at baseline, 6 weeks and 3 months | | | | | | |
| --- | --- | --- | --- | --- | --- | --- |
| **Scale** | | **No. items** | **α* baseline** | | **α* 6 weeks** | **α* 3 months** |
| EORTC QLQ-C30 | | | | | |  |
|  | Global health status/QoL | 2 | | 0.90 | 0.95 | 0.97 |
|  | Physical functioning | 5 | | 0.88 | 0.88 | 0.89 |
|  | Role functioning | 2 | | 0.87 | 0.95 | 0.95 |
|  | Emotional functioning | 4 | | 0.86 | 0.85 | 0.88 |
|  | Cognitive functioning | 2 | | 0.65 | 0.64 | 0.81 |
|  | Social functioning | 2 | | 0.79 | 0.86 | 0.89 |
|  | Fatigue | 3 | | 0.87 | 0.86 | 0.93 |
|  | Nausea and vomiting | 2 | | 0.58 | 0.52 | 0.79 |
|  | Pain | 2 | | 0.77 | 0.86 | 0.78 |
|  | Dyspnea | 1 | | N.A. | N.A. | N.A. |
|  | Insomnia | 1 | | N.A. | N.A. | N.A. |
|  | Appetite | 1 | | N.A. | N.A. | N.A. |
|  | Constipation | 1 | | N.A. | N.A. | N.A. |
|  | Diarrhea | 1 | | N.A. | N.A. | N.A. |
|  | Financial difficulties | 1 | | N.A. | N.A. | N.A. |
| EORTC QLQ-LMC21 | | | | | |  |
|  | Emotional problems | 4 | | 0.87 | 0.69 | 0.82 |
|  | Nutritional problems | 2 | | 0.79 | 0.74 | 0.89 |
|  | Fatigue | 3 | | 0.91 | 0.90 | 0.91 |
|  | Pain | 3 | | 0.74 | 0.75 | 0.74 |
|  | Problems with taste | 1 | | N.A. | N.A. | N.A. |
|  | Dry mouth | 1 | | N.A. | N.A. | N.A. |
|  | Sore mouth/tongue | 1 | | N.A. | N.A. | N.A. |
|  | Peripheral neurophathy | 1 | | N.A. | N.A. | N.A. |
|  | Jaundice | 1 | | N.A. | N.A. | N.A. |
|  | Contact with friends | 1 | | N.A. | N.A. | N.A. |
|  | Talking about feelings | 1 | | N.A. | N.A. | N.A. |
|  | Sex life | 1 | | N.A. | N.A. | N.A. |

* Cronbach’s alpha.

N.A.: not applicable since Cronbach’s alpha can only be determined for scales with multiple items.

| Table S3. Quality of life (median, IQR (25^th^ and 75^th^ percentile)) | | | | | | | |
| --- | --- | --- | --- | --- | --- | --- | --- |
| **Scale** | **Baseline** | **1 week** | **6 weeks** | **3 months** | **6 months** | **9 months** | **12 months** |
|  | *N=50* | *N=26* | *N=49* | *N=49* | *N=18* | *N=9* | *N=3* |
| **QLQ-C30** |  |  |  |  |  |  |  |
| GHS | 83 (67-83) | 41 (25-75) | 66 (50-83) | 66 (54-83) | 83 (60-93) | 75 (50-92) | 83 (33--) |
| PF | 93 (85-100) | 43 (20-87) | 87 (67-93) | 87 (67-100) | 87 (67-100) | 77 (57-98) | 67 (47--) |
| RF | 100 (67-100) | 33 (0-100) | 67 (42-100) | 67 (50-100) | 83 (67-100) | 83 (54-100) | 100 (33--) |
| EF | 83 (75-100) | 79 (56-100) | 83 (67-100) | 83 (67-92) | 83 (67-92) | 71 (54-83) | 67 (67--) |
| CF | 100 (83-100) | 83 (67-100) | 100 (83-100) | 100 (83-100) | 100 (67-100) | 83 (58-100) | 100 (67--) |
| SF | 100 (67-100) | 58 (33-83) | 83 (67-100) | 83 (67-100) | 100 (67-100) | 100 (54-100) | 100 (33--) |
| FA | 22 (0-33) | 61 (42-100) | 33 (22-56) | 22 (11-61) | 22 (0-44) | 22 (3-56) | 22 (0--) |
| NV | 0 (0-0) | 33 (13-67) | 0 (0-17) | 0 (0-17) | 0 (0-0) | 0 (0-17) | 8 (0--) |
| PA | 0 (0-17) | 58 (29-71) | 17 (-33) | 17 (0-33) | 0 (0-33) | 8 (0-58) | 0 (0--) |
| DY | 0 (0-33) | 0 (0-33) | 0 (0-33) | 0 (0-33) | 0 (0-67) | 33 (0-33) | 67 (0--) |
| SL | 0 (0-33) | 33 (0-67) | 0 (0-33) | 0 (0-33) | 33 (0-67) | 33 (8-58) | 33 (0--) |
| AP | 0 (0-0) | 33 (33-100) | 0 (0-33) | 0 (0-33) | 0 (0-33) | 0 (0-25) | 0 (0-0) |
| CO | 0 (0-0) | 17 (0-67) | 0 (0-0) | 0 (0-0) | 0 (0-0) | 0 (0-0) | 0 (0-0) |
| DI | 0 (0-33) | 0 (0-0) | 0 (0-0) | 0 (0-33) | 0 (0-33) | 17 (0-33) | 0 (0--) |
| FI | 0 (0-0) | 0 (0-0) | 0 (0-0) | 0 (0-0) | 0 (0-0) | 0 (0-0) | 0 (0--) |
| **QLQ-LMC21** |  |  |  |  |  |  |  |
| LMCNutri | 0 (0-17) | 67 (33-83) | 17 (0-33) | 17 (0-50) | 0 (0-33) | 0 (0-17) | 0 (0--) |
| LMCFati | 11 (0-33) | 67 (42-92) | 33 (11-44) | 22 (0-61) | 22 (0-44) | 22 (11-69) | 11 (0--) |
| LMCPa | 0 (0-14) | 44 (22-67) | 22 (0-33) | 22 (0-33) | 11 (0-33) | 17 (11-50) | 22 (0--) |
| LMCEp | 25 (17-42) | 42 (17-58) | 25 (17-38) | 25 (17-42) | 33 (17-42) | 21 (17-48) | 17 (17--) |
| LMCWL | 0 (0-0) | 0 (0-0) | 0 (0-17) | 0 (0-0) | 0 (0-33) | 0 (0-33) | 0 (0-0) |
| LMCTA | 0 (0-0) | 0 (0-67) | 0 (0-17) | 0 (0-33) | 0 (0-0) | 0 (0-25) | 0 (0-0) |
| LMCDM | 0 (0-33) | 0 (0-42) | 0 (0-33) | 0 (0-33) | 0 (0-33) | 0 (0-67) | 33 (0--) |
| LMCSM | 0 (0-0) | 0 (0-0) | 0 (0-0) | 0 (0-0) | 0 (0-0) | 0 (0-0) | 0 (0--) |
| LMCPN | 0 (0-33) | 0 (0-33) | 0 (0-33) | 0 (0-33) | 0 (0-33) | 0 (0-33) | 33 (0--) |
| LMCJ | 0 (0-0) | 0 (0-0) | 0 (0-0) | 0 (0-0) | 0 (0-0) | 0 (0-0) | 0 (0--) |
| LMCFr | 0 (0-0) | 33 (0-67) | 0 (0-0) | 0 (0-16) | 0 (0-33) | 0 (0-33) | 0 (0--) |
| LMCFeelings | 0 (0-0) | 0 (0-0) | 0 (0-0) | 0 (0-33) | 0 (0-33) | 17 (0-33) | 0 (0--) |
| LMCSx | 0 (0-33) | 0 (0-67) | 33 (0-33) | 33 (0-67) | 0 (0-58) | 50 (0-67) | 0 (0--) |
