# Supplementary figures and images for: Quality of life in patients with liver tumors treated with holmium-166 radioembolization
Source: Clin Exp Metastasis. 2019 Nov 15;37(1):95–105. doi: 10.1007/s10585-019-10006-1 (PMC7007912; doi:10.1007/s10585-019-10006-1)

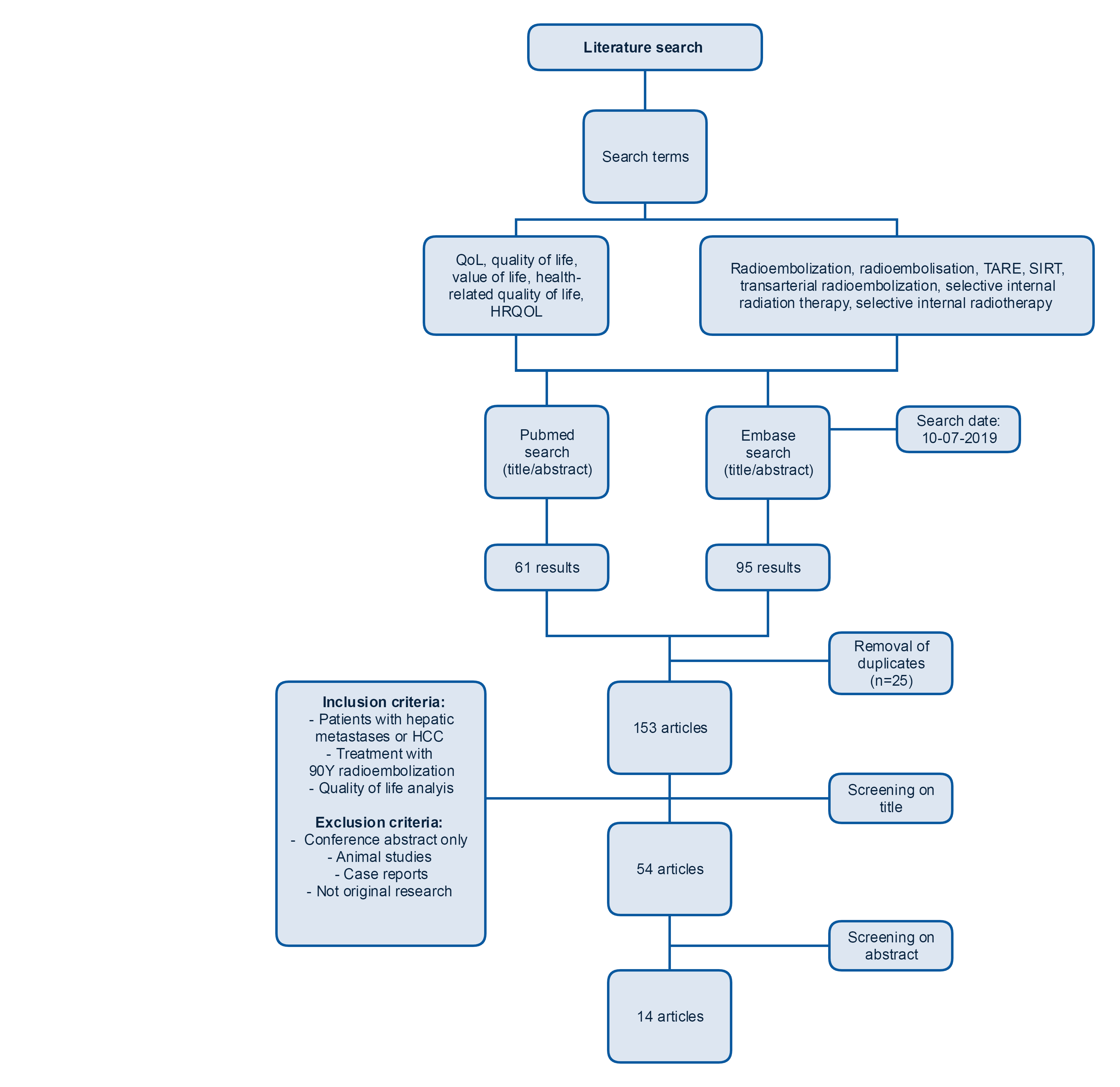

Supplement: Supplementary file 3 — Search strategy for literature review of quality of life studies in patients treated with RE (TIFF 39490 kb) [file 10585_2019_10006_MOESM3_ESM.tif]

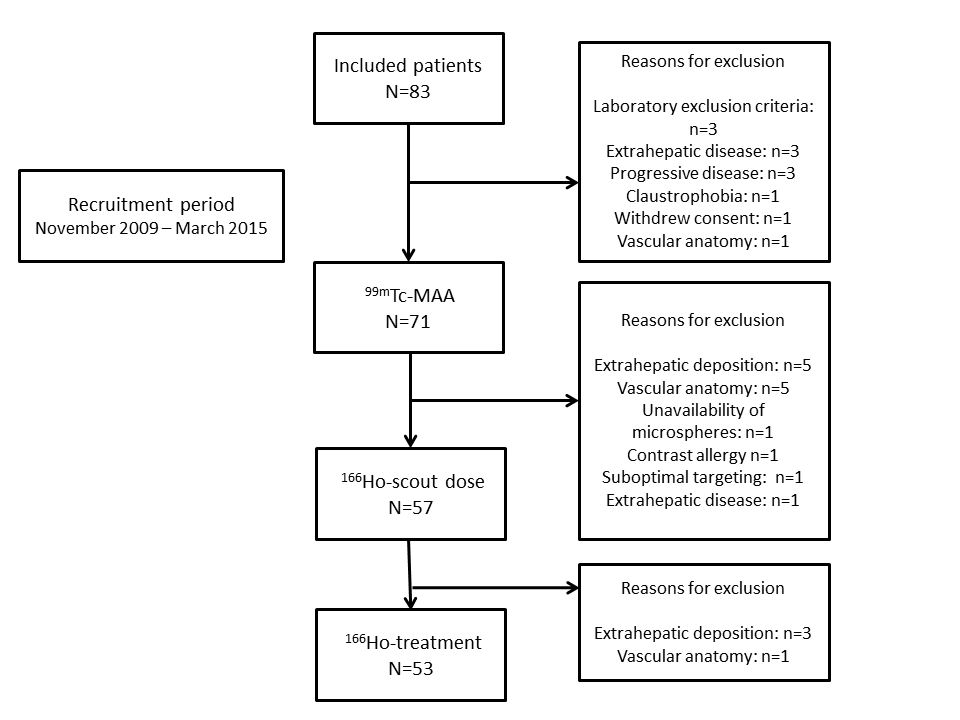

Supplement: Supplementary file 4 — Flowchart of included patients in the HEPAR I and II studies (TIFF 54 kb) [file 10585_2019_10006_MOESM4_ESM.tif]
